# Associations between shade tolerance and wood specific gravity for conifers in contrast to angiosperm trees: Foundations of the conifer fitness‐enhancing shade tolerance hypothesis

**DOI:** 10.1002/pei3.10131

**Published:** 2023-12-13

**Authors:** Gabriel F. Tucker, Douglas A. Maguire, Frederico Tupinambá‐Simões

**Affiliations:** ^1^ Department of Forest Engineering, Resources, and Management Oregon State University Corvallis Oregon USA; ^2^ O'Brien Forest Westport County Mayo Ireland; ^3^ University Institute for Research in Sustainable Forest Management Universidad de Valladolid|UVa Palencia Spain

**Keywords:** drought tolerance, modulus of elasticity, modulus of rupture, polytolerance, relative stiffness, relative strength, shade tolerance, static bending, wood density, wood specific gravity

## Abstract

For decades, researchers have held that wood specific gravity was an indicator or surrogate for both shade tolerance and successional status. However, recent research in dry tropical forests has shown very different associations regarding wood specific gravity. Past analyses of the tolerance and wood properties of tree species have focused on pooled coniferous and angiosperm species in temperate regions; fewer analyses have been conducted separately for conifers and angiosperm species. A database was compiled for the wood properties and/or tolerance scores of 542 temperate Northern Hemisphere conifer and angiosperm trees. Plant strategy was defined by shade tolerance (*T*_shade_), drought tolerance (*T*_drought_), and polytolerance (*T*_poly_ = *T*_shade_ + *T*_drought_) and fundamental wood properties were represented by basic specific gravity (SG_basic_), relative stiffness (MOE/SG_basic_), and relative strength (MOR/SG_basic_). Simple linear regressions tested the significance (*p* < .05) of correlations between plant strategy and wood properties. Conifers, unlike angiosperm trees, showed a negative correlation between *T*_shade_ and SG_basic_ and a positive correlation between *T*_shade_ and both MOE/SG_basic_ and MOR/SG_basic_. Only angiosperm trees had a significant correlation between *T*_poly_ and both SG_basic_ and MOE/SG_basic_, but both conifers and angiosperm trees had a significant correlation between *T*_drought_ and both SG_basic_ and MOE/SG_basic._ Shade tolerance, as a plant strategy, has functional implications for wood properties in temperate Northern Hemisphere conifers but not in associated angiosperms. The implied functional link between wood properties (SG_basic_) and shade tolerance hypothetically extends to other fitness‐enhancing traits impacted by SG_basic,_ such as growth rates and species maximum height.

## Graphical abstract

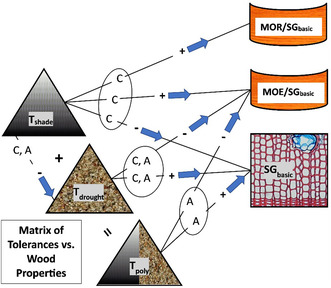

## 1 INTRODUCTION

Shade tolerance is a plant strategy (Reich et al., 2003) or evolutionary pathway with profound implications for the function and structure of plants (Reich et al., 2003) and, in turn, ecosystem structure and dynamics (Franklin et al., 2018; Oliver & Larson, 1996). During recent decades, there has been debate about interpretations of forest stratification relative to forest succession, specifically regarding the relative prevalence of “initial floristics” and “relay floristics” (Egler, 1954; Franklin et al., 2018; Oliver & Oliver, 2018). Regardless, in both scenarios of stand development, dominant overstory trees change with time, and this change is driven to a large extent by the relative shade tolerance of species available at a given site, with species that are more shade tolerant replacing those that are less so. Silviculturists, as applied forest ecologists, rely heavily on a working knowledge of shade tolerance in the design of prescriptions and silvicultural systems for long‐term management of forest ecosystems (Nyland, 2016; Oliver & Larson, 1996; Smith et al., 1997). For many decades, it was also thought that basic wood specific gravity (SG_basic_), also known as green wood density or green wood specific gravity (Bergman et al., 2010; Williamson & Wiemann, 2010), was a functional trait that was so closely linked to shade tolerance that it could be applied as a surrogate for both the shade tolerance of a species and its seral niche in stand dynamics. Following this reasoning, it seemed logical that fast‐growing, fugitive, early seral species (Bazzaz, 1979) would have low SG_basic_, while late‐successional species would have higher SG_basic_ and be slower growing in their shady environment. This notion was based on extensive work in tropical forests (see Augspurger, 1984), but also in temperate forests (Horn, 1971; Williamson, 1975), and recently in the neotropics (Feng et al., 2018). However, Lohbeck et al. (2013) working in tropical forests in southern Mexico showed that early successional species in wet forests did have acquisitive traits, including less dense wood associated with faster growth, and that late‐successional species had conservative traits, including denser wood, but that the opposite was true for species in dry forests. Poorter et al. (2019) analyzed a larger pan‐tropical dataset to demonstrate similarly that the larger collection of species pooled globally for dry and wet tropical forests has opposite trends in SG_basic_ over successional or developmental stages but that average wood density converges to moderate levels of community averages with increasing stand age.

Specific gravity or wood density of a species has also been closely linked to drought tolerance, as denser wood more effectively supports the xylem water conduit, protecting it from cavitation and moisture stress (Chave et al., 2009; Hacke et al., 2001). For any woody plant, wood density is a crucial investment of precious carbon, but perhaps more so for trees that experience intense competition for light. Large woody stems define this tall, massive, perennial life form (Petit & Hampe, 2006) that has independently evolved in arboreal species across many different plant lineages and has provided the foundation of complex forest ecosystem structure. The stiffness and strength that are conferred by tree investment in wood density underscore three crucial roles of woody stems in the evolutionary biology and fitness of tree species: (1) support and orientation of the carbon‐fixing canopy; (2) overtopping and outgrowing competitors; and (3) safe transport of water and solutes (Givnish, 1995).

The relative shade tolerance of a species is generally associated with a complex of characteristics that pertain to its ecophysiology (Valladares & Niinemets, 2008). Adaptations by tree species to shade are particularly evident in the leaf morphology of broadleaf angiosperm species as opposed to conifers (Abrams & Kubiske, 1990). At least since Plinius (Plinius, c A.D. 23–79), humans have been observing the nature of shadow cast by different trees and the varying responses by different understory species (Plinius Secundus, 1885). By the early twentieth century, “tolerance tables” of tree species from North America and Eurasia ranked the ability of different tree species to tolerate shade (Busgen & Munch, 1929; Zon & Graves, 1911). Baker (1949) conducted a survey of many North American academics and practicing silviculturists to reconcile differences between multiple tolerance tables for the region. These revised tables were then used and cited for several decades (e.g., Pacala et al., 1996). Regression analysis of several different rankings and scoring techniques was later used to attach quantitative scores to categorical and subjective classifications of shade, drought, and waterlogging tolerance for 806 Northern Hemisphere woody species (Niinemets & Valladares, 2006). This latter effort allowed for more systematic use of these tolerance scores in the field, as well as for the type of statistical analyses conducted herein.

To more objectively quantify species performance in forest understories and better assign shade tolerance scores, the photosynthetic compensation point of a species has sometimes been employed. This level of light intensity is sufficient to produce enough photosynthate to offset respiration, resulting in net carbon gain and growth (Bazzaz, 1979). Although light compensation point is definitely related to shade tolerance (Franklin et al., 2018), those measuring whole‐plant light compensation point (WPLCP) in comparison to shade tolerance have achieved only mixed results. For example, Baltzer and Thomas (2007) found that species responses to light in seedlings and saplings of eight temperate deciduous angiosperm tree species did not correspond well with their known values of shade tolerance, even while controlling for nutrients in a controlled environment. Lusk and Jorgensen (2013) determined WPLCP by measuring the relative growth rate of the main stem based on the height and basal diameter of a sample of five temperate evergreen species, including three conifers of the Podocarpaceae, growing at varying levels of light intensity in forest understories. They inferred that WPLCPs corresponded well with the published values of shade tolerance for each species but WPLCP was regarded as a less reliable indicator in deciduous forests where the year‐to‐year light regimes would be much more variable. Baltzer and Thomas (2007) also concluded that other traits linked to metabolic costs and seed size, in addition to WPLCP, affect the shade tolerance of a species.

In contrast to shade tolerance, drought tolerance ranks of different species have been derived purely from the moisture‐based climatic conditions characterizing the range (realized niche) of the species. These include total precipitation and potential evapotranspiration (Niinemets & Valladares, 2006).

Since Niinemets and Valladares (2006) assigned comparable quantitative scores to shade and drought tolerance and elaborated on the trade‐off between the two, several investigators have built on their work (Grubb, 2016; Laanisto & Niinemets, 2015; Stahl et al., 2013; Wei et al., 2019). Stahl et al. (2013) analyzed a large plant trait database with nonparametric statistics to develop a whole‐plant spectra of 305 North American woody species, including 97 conifers. Broad trends between conifers and angiosperms revealed distinctly different trait trade‐offs between shade and drought tolerance in these two species groups. Wei et al. (2019) used the tolerance scores developed by Niinemets and Valladares (2006) in a multivariate cluster analysis to classify a broad range of species into two functional groups, that is, tolerators or avoiders of stress. Laanisto and Niinemets (2015) took polytolerance (*T*_shade_ + *T*_drought_) and examined how it relates to waterlogging and cold tolerance. They found that conifers, overall, had lower polytolerance than woody angiosperms but that they could better tolerate both lack of light and water if other stressors such as cold and waterlogging were favorable. In this paper, we assess a bivariate concept of polytolerance (combined shade and drought tolerance; Laanisto & Niinemets, 2015) as an additional plant strategy that can be tested for its statistical and hypothesized functional relationship to wood density.

Denser wood, with a higher carbon investment per unit volume, is both stronger and stiffer, but to differing degrees for different species. Average basic specific gravity, SG_basic_, is also closely associated with the average stiffness and strength of wood from a given species (Jagels et al., 2018), so it is presumably related to drought tolerance and its functional role in stand dynamics (as dictated by shade tolerance). Both wood stiffness and strength are determined by static bending tests (Bergman et al., 2010). Niklas and Spatz (2010) found that while there are strong correlations for both wood strength and stiffness with SG_basic_, the coefficient of variation of the regression consistently increases with increasing SG_basic_ for both conifers and angiosperms.

Serendipity played an important role in the origin of this research, as it likely has in other scientific investigations (Beauchamp, 2019). During the 1990s, when the first author of this paper was initiating applied research into uneven‐aged silviculture (Maguire, 2005; O'Hara, 2014) in conifer forests of the Pacific Northwest, land managers expressed a consistent frustration, that is, that shade‐tolerant conifers amenable to uneven‐aged management, such as western hemlock (*Tsuga heterophylla*) and true firs (*Abies* spp.), were less valuable to the timber industry in part because their lower‐density wood conferred lower strength. From this operational reality arose the overall objective of this study, which is to thoroughly analyze the associations between shade tolerance and wood density in conifers and how they compare to those for associated angiosperm trees. To accomplish this objective, we chose to use not only shade tolerance but also the closely associated plant strategy of drought tolerance and their composite plant strategy of polytolerance. We also used the explanatory variables of relative stiffness and strength of wood, in addition to using specific gravity, as a measure of wood density.

A problem analysis was then used to put the overall objective of the study into the context of the recent research findings discussed above. Hence, we considered the trends previously identified in average wood density of various species across the gradient in tropical seral stages or shade tolerance (Lohbeck et al., 2013; Poorter et al., 2019). If these trends are consistent for a collection of temperate tree species, and if they are consistent for conifers and angiosperms, then this relationship merits further investigation about causal mechanisms driving functional links between wood attributes and shade tolerance. Also, if the trends are consistent for only conifers or only angiosperms, then the reason for this difference among temperate groupings likewise merits further investigation. Finally, if the trends are consistent for only temperate conifers or temperate angiosperms in contrast to no trends or opposite trends in tropical species, then the reason for this difference again merits further investigation.

**FIGURE 1:**
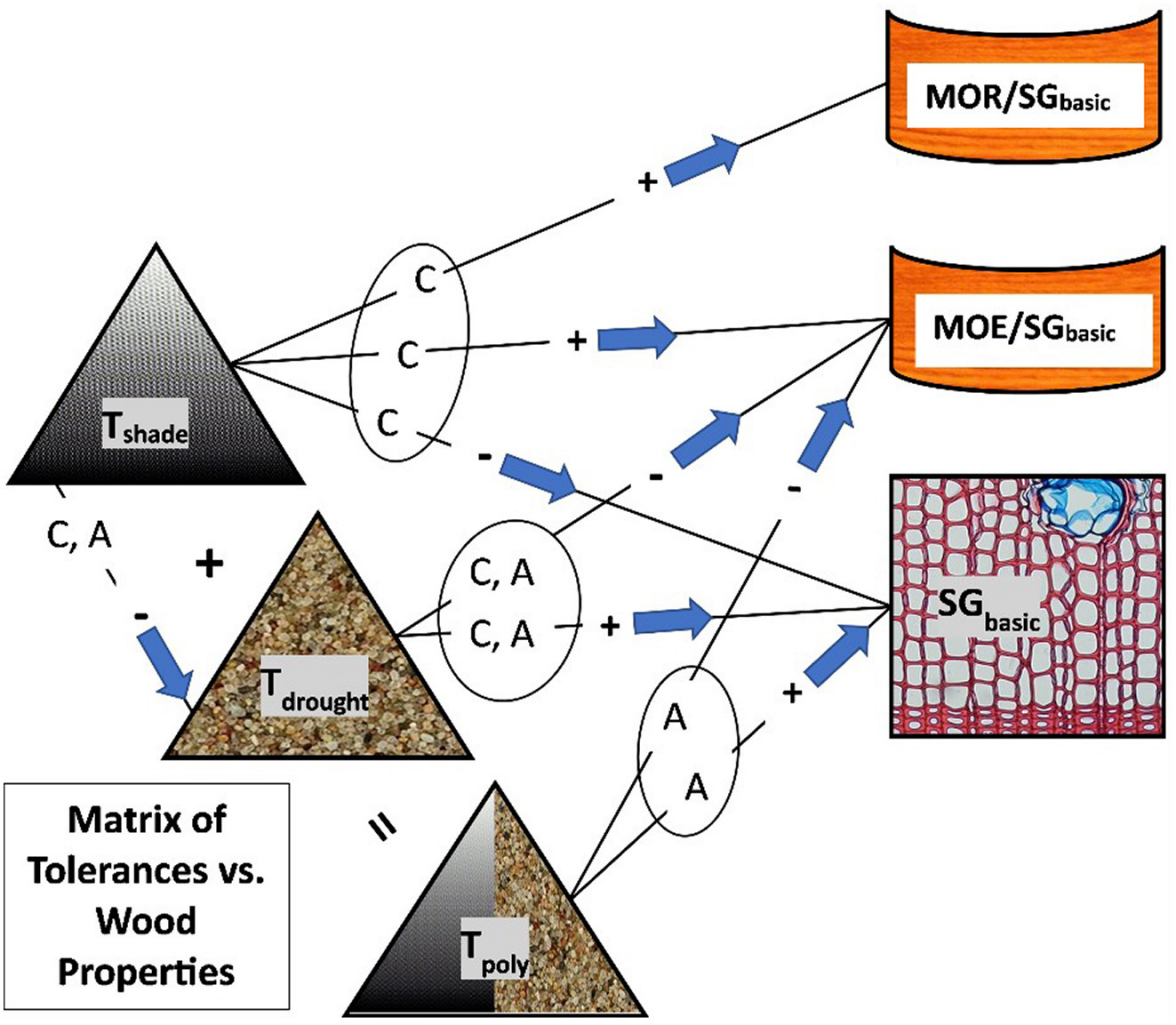
Flowchart of relationships affecting wood function and structure for conifers versus angiosperm trees. Arrows followed by + or − signs indicate statistically significant (*p* < .05) positive or negative correlations, respectively, pointing from the independent to the dependent variable for conifers (*C*) or angiosperm trees (*A*). Counter‐clockwise from upper‐left, relationships begin involving the key plant strategies of shade tolerance (*T*_shade_), drought tolerance (*T*_drought_), and polytolerance (*T*_poly_), which is equal to the sum of *T*_shade_ + *T*_drought_. On the right, wood properties are defined by basic wood specific gravity for green or live wood (SG_basic_) and also static bending variables that are divided by SG_basic_ to yield the relative modulus of elasticity or relative stiffness (MOE/SG_basic_) and the relative modulus of rupture or relative strength (MOR/SG_basic_). Note that relationships involving *T*_shade_ are only significant for coniferous species, while the same is true for *T*_poly_ and angiosperms, and both taxa had significant correlations involving *T*_drought_ versus SG_basic_ and relative stiffness (MOE/SG_basic_).

The pervading null hypothesis for this study, therefore, is that trends in wood density, relative stiffness, and relative strength along gradients in shade, drought, and polytolerance endure widely across both Northern Hemisphere temperate coniferous and angiosperm trees. Given this as our overarching hypothesis, the following nine null hypotheses were tested separately for conifer and angiosperm trees (see Figure 1). That is, there is no statistically significant linear relationship between: (1) *T*_shade_ and SG_basic_, (2) *T*_shade_ and MOE/SG_basic_, (3) *T*_shade_ and MOR/SG_basic_, (4) *T*_drought_ and SG_basic_, (5) *T*_drought_ and MOE/SG_basic_, (6) *T*_drought_ and MOR/SG_basic_, (7) *T*_poly_ and SG_basic_, (8) *T*_poly_ and MOE/SG_basic_, and (9) *T*_poly_ and MOR/SG_basic_. So, at this stage, a total of 18 hypotheses were to be tested, the above nine for both conifers and angiosperm trees. Then, additionally, if neither conifer nor angiosperm trees showed a statistically significant association regarding any one of the above null hypotheses, then the data sets were combined. Thus increasing the sample size of the regression analysis to ensure that, by doing so, the results would not be changed if the regression was generalized for both conifers and angiosperm trees. So, an additional null hypothesis was potentially tested for the comprehensive pool of species. Thus, there were a total of 18 to 27 potential null hypotheses tested for the two taxa.

One other major objective of this research involved developing a working hypothesis about other functional wood attributes that may be indicated by wood density and that therefore represent additional wood traits correlated with relative shade tolerance. Practically speaking, addressing this final objective would lead to a conceptual model about the necessary ecophysiological links between wood traits and shade tolerance. These traits would presumably be selected to ensure reproductive success/fitness through the many complex competitive interactions that are occurring over the course of forest stand development.

## 2 MATERIALS AND METHODS

### 2.1 Data compilation

Data were first compiled into two North American databases of species‐specific variables, one for coniferous trees and one for angiosperm trees. Variables included all three tolerance scores (shade, drought, and polytolerance) and all three wood properties (SG_basic_, MOE/SG_basic_, and MOR/SG_basic_; Figure 1). In addition to the two North American databases that included stiffness and breaking strength, an expanded dataset was compiled to include all species in temperate regions of the Northern Hemisphere for which SG_basic_ was available, thereby maximizing the number of species that contributed to the analysis.

Our primary study area was North America, but limited analyses from the expanded data set provided a wider context. Most North American static bending data (and most of the SG_basic_ data) came from a single laboratory (Bergman et al., 2010). Temperate North America has a relatively diverse array of both conifers and angiosperm tree species, many of which were included in the shade and drought tolerance metric developed by Niinemets and Valladares (2006). Basic specific gravity (SG_basic_) for an additional seven North American coniferous species and corresponding sources for the entire dataset are contained in the data paper published for this publication by Zenodo.org (see Data Availability Statement section below).

**FIGURE A1:**
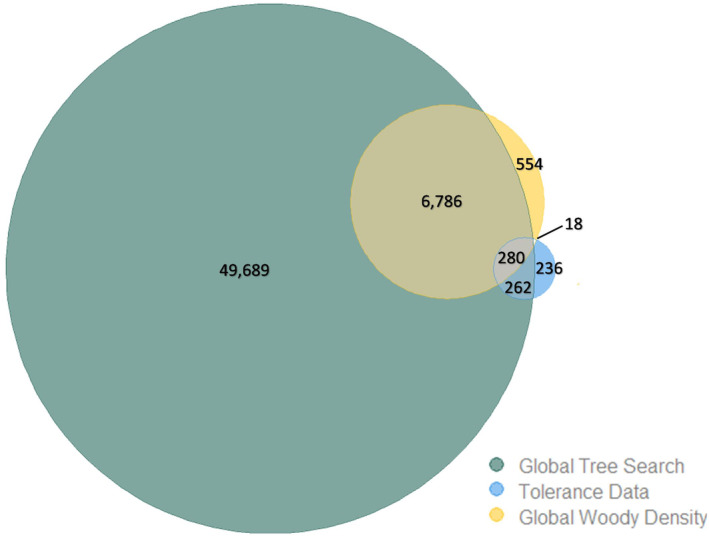
Venn diagram of the merging of the three circles representing databases, each including both conifers and angiosperm tree species, after each was standardized with the WorldFlora R package (Kindt, 2020). The area of three circles and each of the resulting polygons are proportional to WorldFlora standardized taxa numbers, and the polygons are labeled accordingly. For example, 18 nontree species had tolerance and wood density data but were not used in the Northern Hemisphere analysis, while a total of 542 taxa (280 + 262) were used in the analyses shown in Figure A3a,b and 280 taxa were used in the wood density analyses shown in Figure A3c,f. Note the small size of the tolerance data relative to the Global Wood Density and Global Tree Search databases.

All data for the temperate Northern Hemisphere were assembled, standardized, and merged using R (Ihaka & Gentleman, 2016; Kindt, 2020; R Core Team, 2022). Prior to merging these data sets, the scientific names in each of these three databases were separately standardized (see Table A1) using the WorldFlora R package, Version 1.13‐2 (Kindt, 2020). Wood density data originated from the Global Wood Density Database (Chave et al., 2009; Zanne et al., 2009) and was imported into R using the BIOMASS package, Version 2.1.8, for R (Réjou‐Méchain et al., 2017). Tree species were identified using data from Global Tree Search (Beech et al., 2017; BGCI, 2022). Finally, shade and drought tolerance (*T*_shade_ and *T*_drought_) data were sourced by using the entire database for these variables published by Niinemets and Valladares (2006), with the exception of data for *Gingko biloba*, which is a gymnosperm but not a conifer. The merging of these three data sources produced a data set of 280 Northern Hemisphere tree species (both conifer and angiosperm trees; see Table A1 and Figure A1 for sample sizes) with both wood density and tolerance data and another 262 species with only tolerance data. See Venn diagram in Figure A1, which was produced using the R package VennDiagram (Chen & Boutros, 2011).

Wood density is technically defined as the average quotient of weight over volume for a given species and commonly expressed as g/cm^3^ (Chave et al., 2009; Zanne et al., 2009). However, more commonly, the specific gravity of fresh or green wood, known as basic specific gravity (SG_basic_) or green wood specific gravity, has been tabulated (Williamson & Wiemann, 2010). This attribute is defined as the nondimensional ratio of wood density to the density of water, equal to 1.000 at 4.4°C. Green wood density is used to calculate SG_basic,_ and it has traditionally been calculated as the quotient of the oven dry weight of the wood sample divided by its green or fresh volume (Williamson & Wiemann, 2010). Relative strength (MOR/SG_basic_) is based on the modulus of rupture (MOR), a variable of force that is a measure of wood strength in kilopascals (kPa) at the elastic limit of the wood (Bergman et al., 2010). Relative stiffness (MOE/SG_basic_) is measured by the modulus of elasticity (MOE), a measure of the force (MPa) required to produce a given displacement of the wood (Markwardt & Wilson, 1935). As MOE increases, stiffness increases and elasticity declines. We examined the ratio of stiffness and strength to SG_basic_ (MOE/SG_basic_ & MOR/SG_basic_) to standardize the wood attributes relative to SG_basic_ (Jagels et al., 2018).

### 2.2 Statistical analyses

Linear correlations (Snedecor & Cochran, 1980) were computed for both conifer and angiosperm taxa using tolerance scores (*T*_shade_, *T*_drought,_ or *T*_poly_) as the independent variables and wood properties (SG_basic_, MOR/SG_basic,_ or MOE/SG_basic_) as the dependent variables. These correlations were computed separately for conifer and angiosperm species. The correlations between *T*_shade_ and *T*_drought_ were also determined to provide insight into the relative functional independence of the two tolerances, particularly as they impact polytolerance (*T*_poly_). Associations between wood density and relative stiffness and relative strength were not tested because MOR/SG_basic_ and MOE/SG_basic_ included the potential response variable, SG_basic_. All correlations were tested at an *α*‐level of .05 (*p* ≤ .05). If the separate analyses for conifer and angiosperm trees yielded no significant correlation for a particular combination of tolerance and wood property, then the data for the two taxa were combined and the correlation was re‐computed. This was done, as we said in the introduction above regarding the hypotheses, to ensure that the association was not significant for the expanded data set, including both conifers and angiosperm trees.

Two outliers (*Juniperus osteosperma* and *Taxus brevifolia*) were identified in the conifer regression of *T*_shade_ versus SG_basic_ (Figure 3a) because they are clearly farther from the regression line that they contributed to than any of the other data points in that plot. For reference, they were labeled in the other appropriate conifer figures in the North American analysis and their relative positions in the plots discussed as results. Also, four widely distributed species were identified in the North American conifer analysis for reference purposes. Two of these widely distributed species, *Tsuga heterophylla* and *Abies grandis*, are quite shade tolerant but also known to have wood of lower density and are less favored by the timber industry for sawlog production. While the two other widely distributed species, *Pseudotsuga menziesii* and *Picea sitchensis*, are less shade tolerance but with relatively higher wood density and therefore are often favored for sawlog production. All four species are not only ecologically important as native species in the Pacific Northwest (Minore, 1979), but also well adapted and suitable for planting as introduced species in much of the United Kingdom and Ireland (Horgan et al., 2003).

The emphasis of this research is on identifying hypothetical associations between plant strategies related to shade and drought tolerance and structural characteristics related to wood density. Our intention is, therefore, not to find the best fit of a linear or nonlinear regression line as a predictive model but instead to test each null hypothesis of no significant correlation while visually screening for blatant nonlinearity that would limit the utility of statistical tests. The results of the hypothesis tests then guide interpretation relative to more directed future research concerning plant fitness. The *p*‐values and the Pearson correlation coefficients were therefore chosen as the most appropriate statistics to report, as Longuetaud et al. (2016) did in their research on tree wood density relationships in conifers and angiosperm tree species.

**FIGURE A2:**
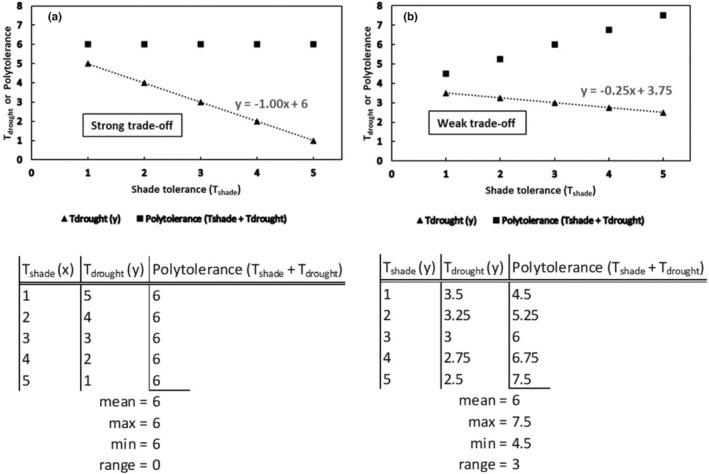
Theoretical comparison of strong (a) and weak (b) trade‐offs between shade and drought tolerance and their respective impacts on polytolerance. (a) slope = −1.0, with maximum trade‐off between shade and drought tolerance, resulting in no change in polytolerance; (b) slope = −.25, with minimal trade‐off between shade and drought tolerance, resulting in a large increase in polytolerance.

The marginal value of identifying linear relationships between the composite polytolerance measure (*T*_poly_ = *T*_shade_ + *T*_drought_) and wood attributes depends largely on the strength of the correlation between *T*_shade_ and *T*_drought_ and the slope of a corresponding regression line (Figure A2). If the trade‐off between shade and drought tolerance was very strong, then the slope of a regression would be very close to one and the variation around the regression would be close to zero, causing polytolerance to vary a little around six (Figure A2a). However, when there is a weak trade‐off between shade and drought tolerance (Figure A2b), it is caused either by a large mean squared error (MSE) and near orthogonality of the two variables or by a small MSE and slope significantly different from zero but very significantly different from one.

## 3 RESULTS

**FIGURE 2:**
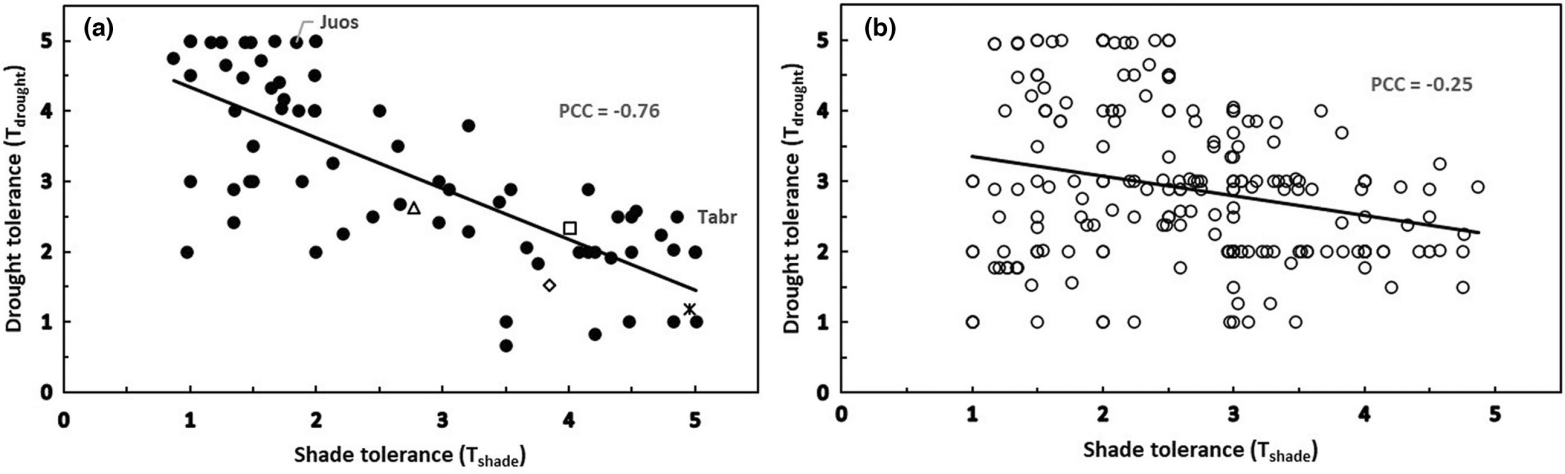
Comparison of trade‐offs between shade tolerance (*T*_shade_) and drought tolerance (*T*_drought_) for North American tree species (PCC = Pearson correlation coefficient): (a) coniferous species; and (b) angiosperm species. The conifers (*n* = 72) have a stronger trade‐off with a slope of −0.72 (intercept = 5.10, *p* < .0001) compared to the woody angiosperm trees (*n* = 211) with a slope of −0.28 (intercept 3.63, *p* = .0003). *Juniperus osteosperma* (Juos) and *Taxus brevifolia* (Tabr) are noted in this figure because they are outliers in Figure 3a. In addition, the following widely distributed species are highlighted in this and other figures concerning North American conifers: *Abies grandis* (open square), *Picea sitchensis* (open diamond), *Pseudotsuga menziesii* (open triangle), and *Tsuga heterophylla* (asterisk). See text and Table A2 for details.

Significant correlations between *T*_shade_ and all three wood attributes (SG_basic_, MOE/SG_basic_, and MOR/SG_basic_) emerged only for conifers, but *T*_shade_ was correlated with no wood attributes in angiosperms (see results summary in Figure 1). In contrast, *T*_drought_ was significantly correlated with SG_basic_ and MOE/SG_basic_ for both coniferous and angiosperm species. The composite tolerance score (*T*_poly_) was correlated with SG_basic_ and MOE/SG_basic_ for only angiosperms. The underlying associations for all of these results are shown in the relatively strong trade‐offs between *T*_shade_ and *T*_drought_ for North American conifers compared to angiosperm tree species (Figure 2a,b). The slope of the *T*_shade_ on *T*_drought_ regression coefficients differed between coniferous and angiosperm species in North America (−.72 compared to −.28, Figure 2a,b). The difference in slope between these two taxonomic species groups had implications for relative polytolerance, as discussed below. The Pearson correlation coefficients also differed between conifer and angiosperm trees (−.76 compared to −.25), illustrating the relative diversity and variability among angiosperm species relative to conifers.

### 3.1 North American conifers

**FIGURE 3:**
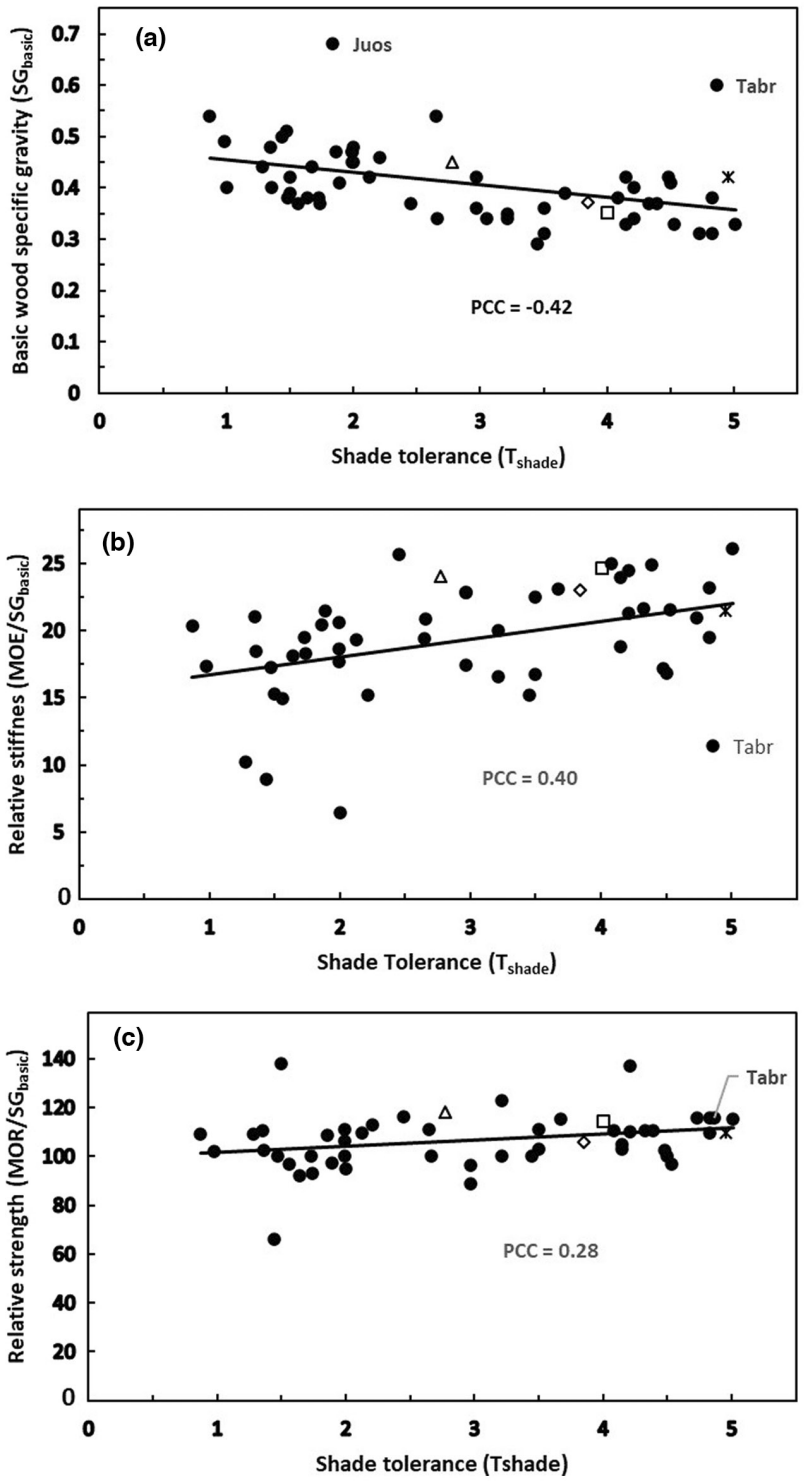
All three significant correlations involving shade tolerance (*T*_shade_) are for conifers alone (PCC = Pearson correlation coefficient). (a) *T*_shade_ versus basic specific gravity (SG_basic_; *n* = 57; *p* = .001). (b) *T*_shade_ versus relative stiffness (MOE/SG_basic_; *n* = 50; *p* = .004). (c) *T*_shade_ versus relative strength (MOR/SG_basic_; *n* = 50; *p* = .045). *Juniperus osteosperma* (Juos) and *Taxus brevifolia* (Tabr) are noted in this figure, where possible in other figures, because they are outliers in plot a above. In addition, the following widely distributed species are highlighted in this and other figures concerning North American conifers: *Abies grandis* (open square), *Picea sitchensis* (open diamond), *Pseudotsuga menziesii* (open triangle), and *Tsuga heterophylla* (asterisk). See the text and Table A2 for details.

**FIGURE 4:**
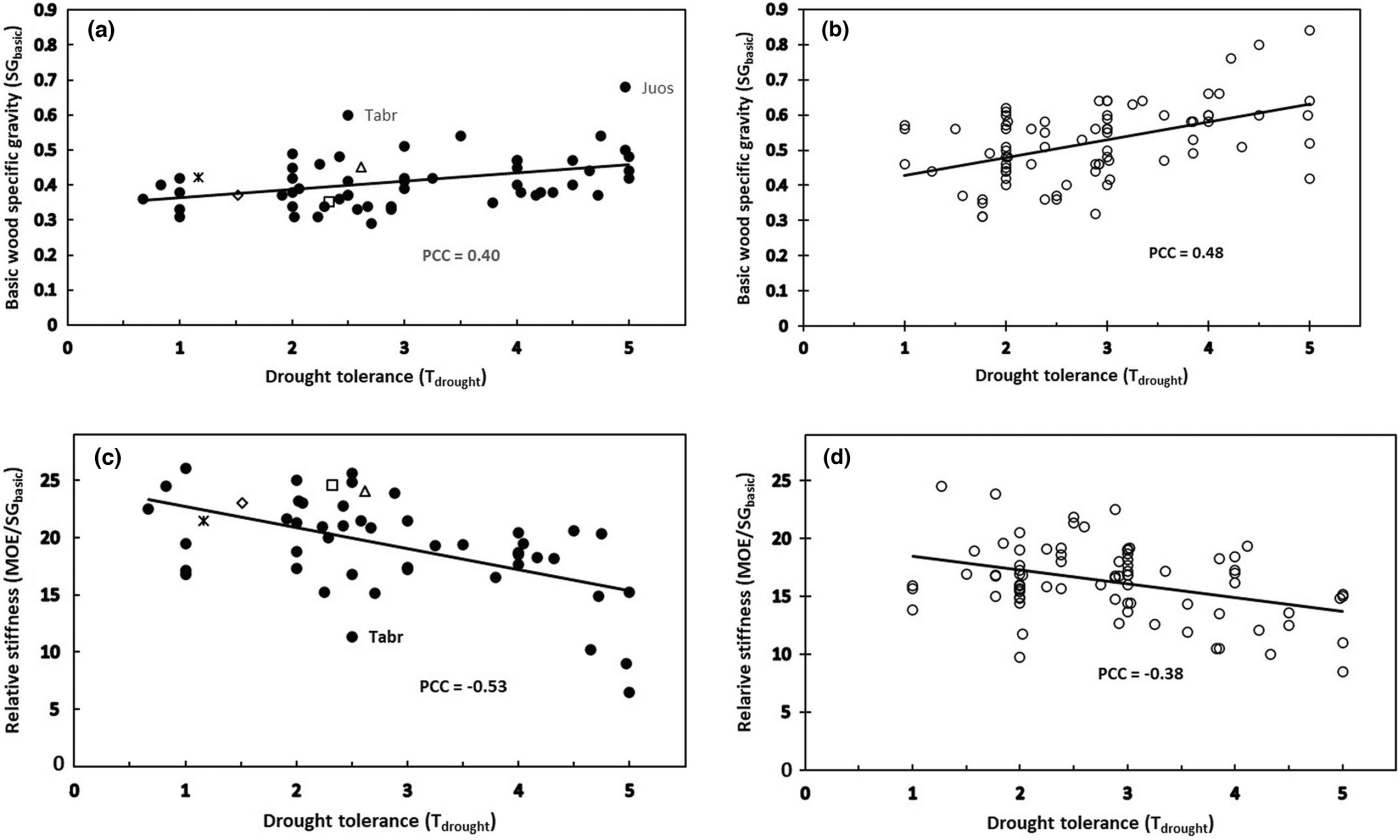
Significant correlations involving drought tolerance (*T*_drought_) were found for both conifers and angiosperm trees (PCC = Pearson correlation coefficient). (a) Conifer *T*_drought_ versus basic specific gravity (SG_basic_; *n* = 57; *p* = .002). (b) angiosperm tree *T*_drought_ versus SG_basic_ (*n* = 79; *p* < 0.0001). (c) Conifer *T*_drought_ versus relative stiffness (MOE/SG_basic_; *n* = 50; *p* < 0.0001). (d) Angiosperm tree *T*_drought_ versus relative stiffness (MOE/SG_basic_; *n* = 79; *p* = .0005). *Juniperus osteosperma* (Juos) and *Taxus brevifolia* (Tabr) are denoted in this figure, where possible, because they are outliers in Figure 3a. In addition, the following widely distributed species are highlighted in this and other figures concerning North American conifers: *Abies grandis* (open square), *Picea sitchensis* (open diamond), *Pseudotsuga menziesii* (open triangle), and *Tsuga heterophylla* (asterisk). See the text and Table A2 for details.

T_shade_ was negatively correlated with SG_basic_ (Figure 3a), which contrasted with the positive correlation reported in the past for selected tropical angiosperm trees (Valladares & Niinemets, 2008). To our knowledge, this negative relationship for conifers has not been previously reported. While *Juniperus osteosperma* and *Taxus brevifolia* appear as outliers, the *p*‐value was still quite low over all species at .001. In contrast to SG_basic_, both MOE/SG_basic_ and MOR/SG_basic_ increased with increasing *T*_shade_ (Figure 3b,c), indicating that as wood density declined with increasing *T*_shade_, strength, and stiffness per unit of density increased. Finally, for conifers, their correlations of *T*_drought_ versus SG_basic_ and MOE/SG_basic_ showed that as wood density increased with increasing *T*_drought_, the stiffness per unit of density decreased (Figure 4a,c).

### 3.2 North American angiosperm trees

The *T*_drought_ results for angiosperm trees (Figure 4b,d) were very similar to those for conifers (Figure 4a,c). However, angiosperm trees did show greater variability in the correlation of *T*_drought_ versus SG_basic_ compared to conifers (compare Pearson correlation coefficients in Figure 4b vs. 4a). The greater variability in SG_basic_ for coefficients in angiosperm trees is even more pronounced in tropical angiosperm species, ranging in SG_basic_ from .05 to 1.0 (Williamson, 1984).

**FIGURE 5:**
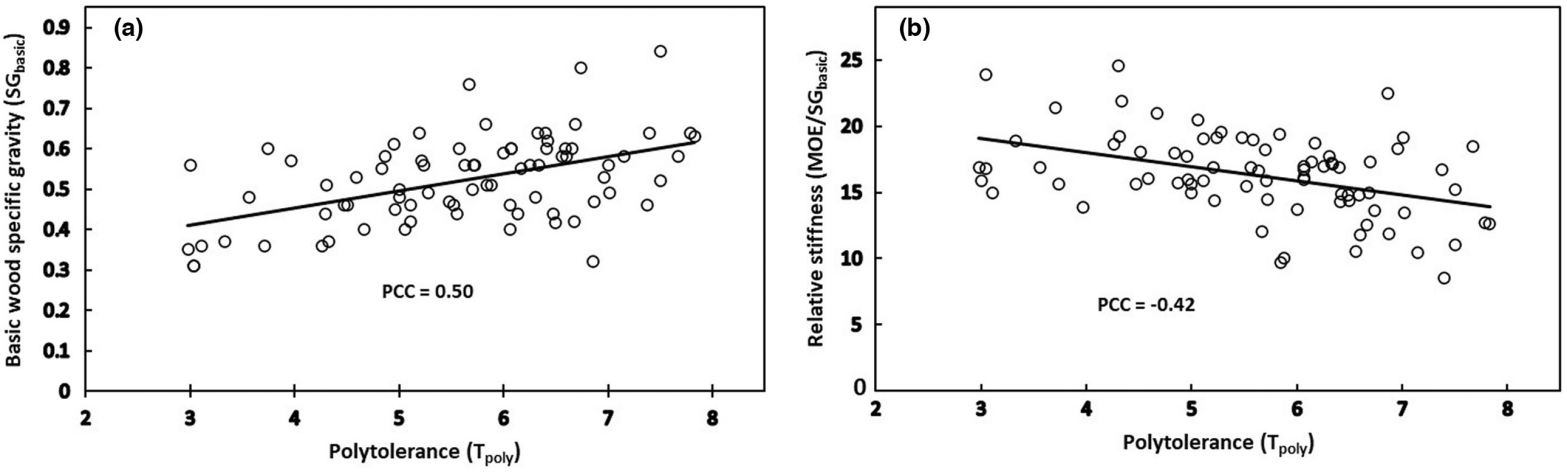
Both significant correlations involving polytolerance (*T*_poly_) are for angiosperm trees alone (PCC = Pearson correlation coefficient). (a) *T*_poly_ versus basic wood specific gravity (SG_basic_; *n* = 79; *p* = 3.27E‐06). (b) *T*_poly_ versus relative stiffness (MOE/SG_basic_; *n* = 79; *p* = 9.99E‐05). See Table A2 for details.

Unlike the results for conifers, *T*_poly_ in angiosperms was strongly correlated with both SG_basic_ and MOE/SG_basic_ (Figure 5). Comparisons of polytolerance scores have been documented in the literature (Laanisto & Niinemets, 2015; Niinemets & Valladares, 2006; Wei et al., 2019); however, to our knowledge *T*_poly_ has not previously been analyzed for its relationship to specific plant traits.

### 3.3 Temperate northern hemisphere species

**FIGURE A3:**
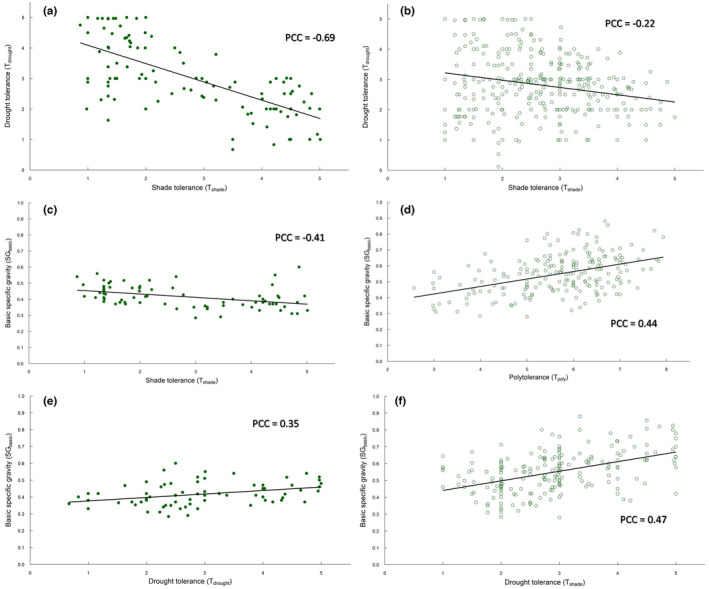
Analysis of temperate Northern Hemisphere tree species regarding shade, drought, and polytolerance (*T*_shade_, *T*_drought_, and *T*_poly_) and basic specific gravity (SG_basic_) data (PCC = Pearson correlation coefficient). (a) *T*_shade_ versus *T*_drought_ of 111 conifer tree species. (b) *T*_shade_ versus *T*_drought_ for 431 angiosperm tree species. (c) *T*_shade_ versus SG_basic_ for 74 conifer tree species (d) *T*_poly_ versus SG_basic_ for 206 angiosperm tree species. (e) *T*_drought_ versus SG_basic_ for 74 conifer tree species. (f) *T*_drought_ versus SG_basic_ for 206 angiosperm tree species. Note: these results are similar to for those from the North American data set; see Figures 2a,b, 3a, 4a,b, and 5a, respectively. See Table A2 for details.

Results from tests involving *T*_shade_ versus *T*_drought_ and SG_basic_ in the expanded temperate Northern Hemisphere dataset (Figure A3) were the same as the results in the North American dataset, and the direction of the correlations were identical (Figures A3a vs. 2a, Figures A3b vs. 2b, Figures A3c vs. 3a, Figures A3d vs. 5a, Figures A3e vs. 4a, Figures A3f vs. 4b). Relationships involving relative wood stiffness and relative wood strength could not be tested because these attributes were not available for all the species in the more comprehensive dataset for temperate Northern Hemisphere species.

## 4 DISCUSSION

The results supported rejection of the primary null hypothesis, specifically that no significant relationships exist between any of the individual tolerance measures (*T*_shade_, *T*_drought_, and *T*_poly_) and any of the individual wood attributes (SG_basic_, MOE/SG_basic_, MOR/SG_basic_), both for coniferous species and for angiosperm species. This rejection of the primary hypothesis reflected the rejection of numerous secondary null hypotheses listed above, including some for conifers and some for angiosperms. However, only the four relationships involving two of the tolerance measures (*T*_drought_ and *T*_poly_) and two of the wood attributes (SG_basic_ and MOE/SG_basic_) were rejected consistently for both conifer and angiosperm tree species. The only significant relationship for MOR/SG_basic_ was with shade tolerance for conifers, but shade tolerance in angiosperm trees did not have any significant relationship to any of the three wood properties. These differences impacted the respective trade‐offs between shade and drought tolerance in these two coarse taxonomic groups (Figure 2; Figure A3a,b; theoretical example in Figure A1), specifically for polytolerance in angiosperm tree species. The importance of polytolerance in angiosperms was at least in part a result of the insignificant correlations between shade tolerance and both SG_basic_ and MOE/SG_basic_. In contrast, the lack of significant correlation between any wood attributes and *T*_poly_ in conifers was driven by the strongly negative (and hence “canceling”) correlation between *T*_shade_ and *T*_drought_ in that taxonomic group. The dispersion of the data points presents a striking difference between conifers and angiosperms with respect to the relationship between *T*_shade_ and *T*_drought_ (Figure 2a,b; Figure A3a,b). Grubb (2016) critiqued the concept of tradeoff between *T*_shade_ and *T*_drought_ (Niinemets & Valladares, 2006) using three data sets for woody plants in North America, Europe, and East Asia. He concluded that in these, as in many other cases, the term “trade‐off” was inappropriate as there were many pairs of data points and therefore species that were not experiencing any trade‐off at all, even if the correlation was statistically significant. He argued that in such cases, a boundary‐line analysis would be more appropriate than a regression analysis. While it is true that the conditional distribution of shade tolerances at a given level of drought tolerance overlaps the conditional distribution at other levels of drought tolerance to a very large degree (Figure 2a,b), the correlations were statistically significant and justified a rejection of the null hypothesis of no significant relationship. Manipulative experiments would be needed for stronger inferences that distinguish between species that have evolved under environmental conditions demanding a tradeoff and those that have evolved with selective pressure for only shade stress or only drought stress. Polytolerance (*T*_poly_) in conifers may not have correlated well with SG_basic_ or MOE/SG_basic_ because of limitations on the structure of their wood (largely just tracheids with only some ray parenchyma) that can confer both shade and drought tolerance. Conversely, the greater structural variation in angiosperm wood (ring‐porous vs. diffuse‐porose, highly variable vessel elements, and larger and more variable rays) may have allowed angiosperms to respond more strongly to selective pressure from both light and drought stress simultaneously.

The light partitioning hypothesis (LPH; Poorter & Arets, 2003) is an approach used in the tropics to better understand and assess the shade tolerance of understory trees and seems to have relevance with regard to this analysis in the temperate zone. The LPH is based, at least in part, on the concept of gap partitioning (Denslow, 2009), and the LPH is defined as being based on the following three premises: “(1) there is a gradient in light availability at the forest floor; (2) tree species show a differential distribution with respect to light; and (3) there is a trade‐off in species performance that explains their different position along the light gradient.” Hence, the above differing trade‐offs for conifers and angiosperm trees, regarding *T*_shade_ and *T*_drought_, also demonstrate the strength of the LPH with these species in the temperate zone. This is because, overall, conifers in particular with high *T*_shade_ tend to have low *T*_drought_ and vice versa. Furthermore, because conifers are evergreen, they are inherently influenced by light for a larger portion of the year and, at high latitudes, light is extremely variable during the year and comes from much lower angles in the sky compared to in the tropics.

**FIGURE A4:**
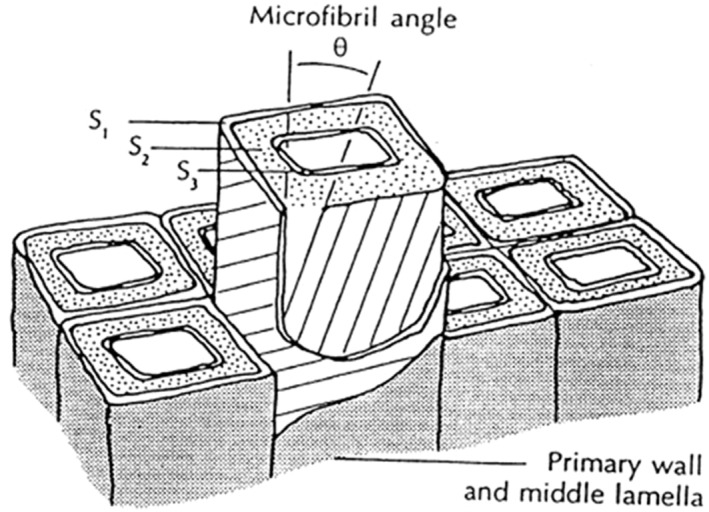
Microfibril angle orientation in the S_2_ cell wall layer of a conifer tracheid (from Dickson & Walker, 1997).

The mechanisms by which increased SG_basic_ enhances drought tolerance (Figure 4a,b) and absolute wood stiffness and strength through carbon investment in wood seem more obvious than how declining SG_basic_ would increase MOE/SG_basic_ and/or MOR/SG_basic_ (Figures 3 and 4). Some clarity can be gained by remembering that the latter result suggests that these variables are the ratio of MOE or MOR to SG_basic_, implying that MOE and MOR do not increase linearly as SG_basic_ increases. Recent work by Jagels et al. (2018) provided evidence that trees worldwide show an increase in relative stiffness and perhaps also relative strength with increasing maximum height. They presented evidence that conifers can improve cellulose quality and adjust tracheid length and microfibril angle (Figure A4) as a strategy for resisting the greater bending stress on taller trees while minimizing vulnerabilities associated with greater weight. These same features may also reduce the risk of cavitation and improve water conduction under stressful conditions of low soil water potential and/or high vapor pressure deficit. Some conifers, such as *Pseudotsuga menziesii*, and certain members of the Taxaceae have spiral thickenings that reinforce vertical tracheids (Kukachka, 1960). The reason that this issue is less clear for angiosperm trees may be that they represent a more diverse taxonomic group with more complex and variable wood anatomy (Jagels et al., 2018). Longitudinal vessels specialize in conduction, while parenchyma cells and fibers provide support. In contrast, conifer wood is dominated simply by tracheids.

Results for conifers showing an increase in SG_basic_ with increasing *T*_drought_ (Figure 4a) were consistent with similar findings in the literature regarding drought stress for all woody plants (Chave et al., 2009; Hacke et al., 2001; Valladares & Niinemets, 2008). While all extremely drought‐tolerant species have dense wood, not all species with extremely dense wood are necessarily very drought tolerant. *Taxus brevifolia*, for example, has relatively dense wood and is extremely shade tolerant (Figure 3a), but only moderately drought tolerant (Figure 4a). This species is rather unique among conifers as a small understory tree with relatively flat planar leaves that perhaps contribute to its extreme shade tolerance but also to its relative susceptibility to water loss and drought stress. This species therefore often experiences high mortality when exposed to direct sunlight in very open conditions, despite its extremely dense wood. While the relationship of conifer *T*_drought_ to MOE/SG_basic_ (Figure 4c) was negative, this relationship is also consistent with the negative correlation of *T*_shade_ to SG_basic_ (Figure 3a) because, in both cases, as wood density increases, stiffness relative to wood density decreases.

The four widely distributed conifer species labeled in their graphs are, for the most part, on the upper end of the shade tolerance scale, with *Pseudotsuga menziesii* having the lowest shade tolerance at *T*_shade_ = 2.78. Indeed, all trees must be more or less shade tolerant due to self‐shading within the canopy, but these four are more so.

The focus of this research is clearly on conifer species and how their associations regarding tolerance and wood density contrast with those of angiosperm trees. Because angiosperm trees are a vastly more diverse group of plants than conifers, future work could examine particular groups of angiosperm trees with particular functional and structural traits, for example, ring porous versus diffuse porous species.

### 4.1 The conifer fitness‐enhancing shade tolerance hypothesis

Shade tolerance is arguably more influential on the evolution of conifer species simply because leaf area index varies less on annual and seasonal scales in evergreen forests, imposing more constant shade on understories, particularly in boreal regions where sun angles are so low (Walker & Kenkel, 2000). Few comparisons are available for year‐round light levels in adjacent conifer and deciduous broadleaf forest types, particularly for species mixes with comparable leaf area index. Messier et al. (1998) reported that, during a growing season in boreal forests in Canada, the photosynthetic photon flux density under the deciduous broad‐leaved stands was more consistent and intense with its diffuse light than under nearby stands of evergreen conifers. Also, using hemispherical photography, Liu et al. (2015) found significantly higher availability of both direct and indirect light under a natural subtropical evergreen hardwood forest than under an adjacent evergreen conifer plantation of *Cryptomeria japonica*. Year‐round light is important to consider when comparing evergreen coniferous forests to deciduous angiosperm forests, the latter of which have a leafless springtime when seed germination and early seedling development can take place. Established understory seedlings in a deciduous broadleaf forest can likewise benefit from significant carbon gain through phenological avoidance of shade. This takes place when understory plants adjust their phenology and leaf out earlier in the spring and/or have leaf senescence later in the autumn than overstory trees (Augspurger et al., 2005). Obviously, seedlings under the canopy of an evergreen coniferous forest cannot benefit from such a process because the overstory canopy is never leafless, illustrating why the evolution of T_shade_ is particularly important for conifers.

**FIGURE A5:**
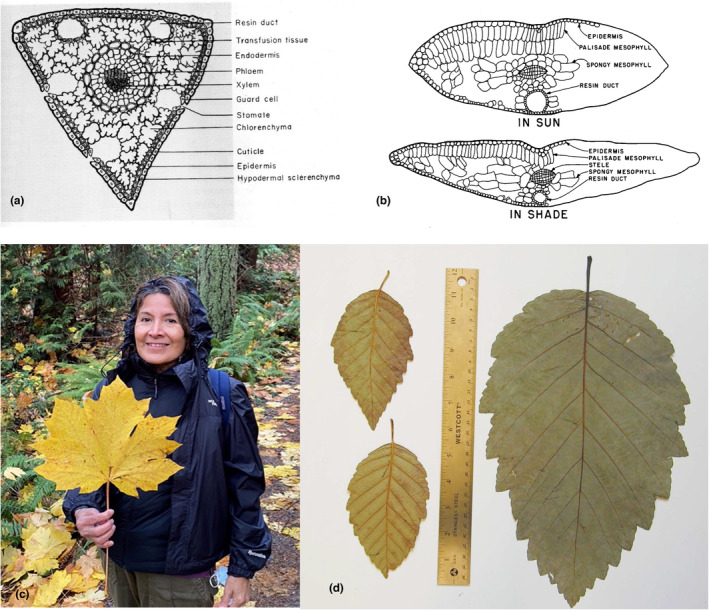
Architectural constraint of conifer leaves, most of which have only a single midvein, in contrast to angiosperm trees with multi‐veined leaves. (a) Several taxa of conifers have very narrow needle‐like leaves with chloroplasts in chlorenchyma or undifferentiated mesophyll, as in *Pinus strobus* (Kozlowski, 1971), with leaves approx. 0.85 mm in width. (b) Certain taxa of conifers do have more planar leaves with most chloroplasts in differentiated palisade mesophyll, particularly those developed in shade, but they are still very narrow with only a single midvein, as with shade leaves of *Tsuga heterophylla*, approx. 1.80 mm in width (Tucker & Emmingham, 1977). (c) In contrast, angiosperm trees have multi‐veined laminar leaves, which can be quite large, as with *Acer macrophyllum*, which has leaves approx. 15–30 cm in width. (d) Certain angiosperm trees also exhibit remarkable plasticity from sun to shade foliage, as with this example of *Alnus rubra*, which has shade leaves with 5.6 times the area of those in the sun (Chan et al., 2003).

Given that the understories of evergreen coniferous forests are darker on a year‐round basis than the understories of comparable deciduous broadleaf forests, we argue that conifers, using a shade‐tolerant strategy, have evolved to economize on their carbon investment and reduce their wood density in more shade‐tolerant species. This is at least in part because they are less adept at improving the efficiency of leaf carbon gain through modified leaf morphology, as has been documented for many broadleaf species (Abrams & Kubiske, 1990; Givnish, 1988; Valladares & Niinemets, 2008). Alternatively, conifers may experience greater selective pressure for more efficient use of the carbon that they can fix. This mechanism may emerge at least partly because conifer needles are more closely bound by architectural constraints (Gould & Lewontin, 1979) or perhaps by trait delimitation (Olson, 2019) and therefore are unable to effectively modify their leaves as an adaptation to shade (see Figure A5).

**FIGURE A6:**
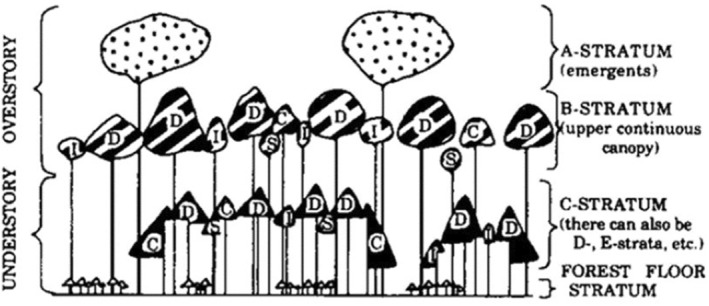
Canopy emergents are in the A‐Stratum and the tallest trees in the forest. Letters denote crown classes (C, codominant; D, dominant; I, intermediate; S, suppressed (overtopped)). The lower stratum potentially contains trees from each crown class (from Oliver & Larson, 1996).

Having a lower wood density should promote the fitness of shade‐tolerant conifers in multiple ways. Essentially, it should put them on the fast end of Reich's (2014) “fast‐slow” plant economic spectrum. Gibert et al. (2016) found that ontogenetic effects or changes driven by plant age and size do not affect correlations of wood density with growth rate. Iida et al. (2012) found that species with low wood density more efficiently expand in height, thus attaining better light conditions. From the perspective of forest ecology and stand dynamics, lower SG_basic_ and increased height growth potential provide an explanation for how, by virtue of their unique form of shade tolerance, many late seral conifers are able to stay relatively healthy in the understory by economizing on wood density. Then, when the canopy does open, they are able to rapidly grow through the upper stratum and succeed as emergents (Figure A6). This growth behavior suggests that conifers, like at least some dry‐forest tropical trees (Lohbeck et al., 2013), are expressing acquisitive traits, not early in succession or stand establishment to capture growing space on open ground, but late in succession or structural development to express dominance and disseminate more pollen and seed in an established forest.

As a final caveat, the vast majority of conifer data in this study is from the Pinaceae and the Cupressaceae, which are the predominant conifer families of the temperate Northern Hemisphere, comprising 61% of all conifer species worldwide (Eckenwalder, 2009). However, other conifer families offer few comparable *T*_shade_ data and therefore need investigation. Two such families, which are also known to have members with relatively high SG_basic_, are the Podocarpaceae (Coomes & Bellingham, 2011) and the Taxaceae (see Section 2 regarding outlier *T. brevifolia* above).

We propose that future research consider a conifer fitness‐enhancing shade tolerance hypothesis (CFEST Hyp.) which is based on the following three premises:

1. Conifer forests impose shade on a year‐round basis
2. Many late‐seral shade‐tolerant conifers of the Northern Hemisphere have evolved with lower wood density, at least in part due to the architectural constraints of needle‐like leaves that influence and impose lower carbon availability
3. Thus, many shade‐tolerant conifers will grow faster and taller as canopy emergents with enhanced fitness compared to early successional shade‐intolerant conifer species.

## 5 CONCLUSIONS

Findings indicate a statistically significant association exists between wood density (SG_basic_) and shade tolerance (*T*_shade_) for Northern Hemisphere conifers, in contrast to angiosperm tree species. Furthermore our findings of decreasing wood density with increasing shade tolerance in conifers are shown to coincide with increasing relative stiffness and strength of wood. Hence, conifers can be seen as economizing on their carbon investment in wood while at the same time improving its function within the tree. These findings support the work in tropical forests by Lohbeck et al. (2013) and Poorter et al. (2019). However, it contrasts with other findings that characterize all late seral shade‐tolerant trees as having relatively dense wood and early seral shade‐intolerant trees as having much lower wood density. Continuing progress on understanding the interplay between *T*_shade_ and wood density will rely on the collection of additional *T*_shade_ data for other species worldwide. In the meantime, the CFEST hypothesis may provide some guidance based on currently available data. Our future research will test the CFEST hypothesis in relation to other fitness‐enhancing traits associated with SG_basic_, such as growth rates and species maximum height. These findings and future research regarding the CFEST hypothesis may improve general understanding and appreciation of shade‐tolerant conifers because, as discussed, several are widely distributed, not just in their native ranges but also in regions where they are planted as introduced species. Finally, if the CFEST hypothesis holds up to the test of operational silviculture and rigorous ecological and silvicultural research, it could encourage expanded exploration of uneven‐aged forest management with shade‐tolerant conifers. Although wood density will in many cases be lower, the potential may also exist for increased growth rates, greater maximum height, increased stand productivity, and longer‐term carbon sequestration.

## FUNDING INFORMATION

None.

## CONFLICT OF INTEREST STATEMENT

The authors declare no conflict of interest.

## Data Availability

All of the data and/or its published sources used in this paper have been provided above or are contained in the related data paper published by the Zenodo data repository at http://doi.org/10.5281/zenodo.10218710, including the R script used in the compilation and standardization of the data on Northern Hemisphere tree species used in Figure A3.

## APPENDIX A

**TABLE A1:** Compilation and Standardization of Northern Hemisphere databases.

Description	Global wood density	Global tree search	Tolerance data	Comments
Database size (number of records)	16,467	57,958	805	*Ginkgo biloba* removed from tolerance data
Duplicate taxa values due to multiple records per taxa	−8,386	—	—	Duplicate records were averaged in
Duplicated values due to synonyms detected by WorldFlora	−443	−941	−9	Duplicate species values averaged in
Total standardized unique taxa (sum of previous rows)	7,638	57,017	796	Proportional to Venn diagram circle area

**TABLE A2:** Regression equations and statistics for all statistically significant relationships.

No.	Fig.	Reg.	A/C	*X*	*Y*	Equation	*n*	*p*	PCC
1	2a	NA	C	*T*_shade_	*T*_drought_	y = −0.7199*x* + 5.0642	72	1.54E‐14	−.756
2	2b	NA	A	*T*_shade_	*T*_drought_	*y* = −0.2806*x* + 3.6333	211	2.76E‐04	−.248
3	3a	NA	C	*T*_shade_	SG_basic_	*y* = −0.0245*x* + 0.4794	57	1.03E‐03	−.423
4	3b	NA	C	*T*_shade_	MOE/SG_basic_	*y* = 1.3287*x* + 15.401	50	3.68E‐03	.403
5	3c	NA	C	*T*_shade_	MOR/Sg_basic_	*y* = 2.514*x* + 99.076	50	4.49E‐02	.285
6	4a	NA	C	*T*_drought_	SG_basic_	*y* = 0.0239*x* + 0.3392	57	1.86E‐03	.403
7	4b	NA	A	*T*_drought_	SG_basic_	*y* = 0.0504*x* + 0.3788	79	7.53E‐06	.480
8	4c	NA	C	*T*_drought_	MOE/SG_basic_	*y* = −1.8365*x* + 24.578	50	7.23E‐05	−.531
9	4d	NA	A	*T*_drought_	MOE/SG_basic_	*y* = −1.1808*x* + 19.629	79	5.31E‐04	−.381
10	5a	NA	A	*T*_poly_	SG_basic_	*y* = 0.0424*x* + 0.2829	79	3.27E‐06	.496
11	5b	NA	A	*T*_poly_	MOE/SG_basic_	*y* = −1.0692*x* + 22.299	79	9.99E‐05	−.424
12	A3a	NH	C	*T*_shade_	*T*_drought_	*y* = −0.5992*x* + 4.6879	111	5.93E‐17	−.689
13	A3b	NH	A	*T*_shade_	*T*_drought_	*y* = −0.2407*x* + 3.4583	431	3.84E‐06	−.220
14	A3c	NH	C	*T*_shade_	SG_basic_	*y* = −0.0212*x* + 0.4751	74	2.93E‐04	−.409
15	A3d	NH	A	*T*_poly_	SG_basic_	*y* = 0.0472*x* + 0.2801	206	3.29E‐11	.441
16	A3e	NH	C	*T*_drought_	SG_basic_	*y* = 0.0205*x* + 0.3563	74	2.20E‐03	.351
17	A3f	NH	A	*T*_drought_	SG_basic_	*y* = 0.0572*x* + 0.3826	206	1.64E‐12	.466

Abbreviations: A/C, angiosperm tree or conifer; NA, North American; NH, Northern Hemisphere; PCC, Pearson correlation coefficient.; Reg., region; *T*_drought_, drought tolerance; *T*_poly_, polytolerance (*T*_shade_ + *T*_drought_); *T*_shade_, shade tolerance.
